# 1587. SOLAR 12-Month North American Results: Randomized Switch Trial of CAB+RPV LA vs. Oral BIC/FTC/TAF

**DOI:** 10.1093/ofid/ofad500.1422

**Published:** 2023-11-27

**Authors:** Mehri McKellar, Paula Teichner, Christopher Bettacchi, Jonathan Angel, Lori A Gordon, Kenneth Sutton, Denise Sutherland-Phillips, Christine L Talarico, Rimgaile Urbaityte, Rodica Van Solingen-Ristea, Bryan Baugh, Ronald D’Amico, Jean A van Wyk

**Affiliations:** Duke University Hospital, DURHAM, North Carolina; ViiV Healthcare, Chicago, Illinois; North Texas Infectious Disease Consultants, Dallas, Texas; Ottawa Hospital Research Institute, Ottawa, Ontario, Canada; ViiV Healthcare, Chicago, Illinois; ViiV Healthcare, Chicago, Illinois; ViiV Healthcare, Chicago, Illinois; ViiV Healthcare, Chicago, Illinois; GSK, Uxbridge, England, United Kingdom; Janssen Research & Development, LLC, Beerse, Antwerpen, Belgium; Janssen Research & Development LLC, Raritan, New Jersey; ViiV Healthcare, Chicago, Illinois; ViiV Healthcare, Brentford, UK, Brentford, England, United Kingdom

## Abstract

**Background:**

Cabotegravir + rilpivirine (CAB+RPV) administered monthly or every 2 months (Q2M) is the only complete long-acting (LA) regimen for maintaining HIV-1 suppression and may address challenges associated with daily oral therapy. In the Phase 3b SOLAR study, switching to CAB+RPV LA Q2M was noninferior to continuing daily oral bictegravir/emtricitabine/tenofovir alafenamide (BIC/FTC/TAF). We present results for North American (NA; United States and Canada) participants.

**Methods:**

SOLAR (NCT04542070) is the first randomized (2:1), open-label, multicenter, noninferiority study assessing switching virologically suppressed adults to CAB+RPV LA Q2M (with oral lead-in or starting with injections) vs. continuing BIC/FTC/TAF. The primary analysis was based on the prespecified modified intention-to-treat exposed (mITT-E) population (n=11 from 1 study site excluded from the ITT-E population for protocol deviation) at Month (M) 12. The primary endpoint was the proportion with plasma HIV-1 RNA ≥50 c/mL. Other endpoints were the proportion with plasma HIV-1 RNA < 50 c/mL, incidence of confirmed virologic failure (CVF; 2 consecutive HIV-1 RNA ≥200 c/mL), safety and tolerability (ITT-E), and treatment satisfaction (HIV Treatment Satisfaction Questionnaire status version [HIVTSQs]).

**Results:**

Of 670 participants (mITT-E), 325 were from North America; 66% (n=216/325) switched to LA and 34% (n=109/325) continued BIC/FTC/TAF. Baseline (BL) characteristics were similar between arms (**Table 1**). At M12, 1 participant in each arm had HIV-1 RNA ≥50 c/mL (**Table 2**). No NA participant had CVF in the mITT-E population; 1 (0.3%) NA participant excluded from the mITT-E population due to protocol violation had CVF (LA arm). Adverse events (AEs), excluding injection site reactions (ISRs), were similar between the LA (74% [n=164/223]) and BIC/FTC/TAF arms (73% [n=83/113]). More participants in the LA vs. BIC/FTC/TAF arm withdrew due to AEs (8% [n=17/223] vs. < 1% [n=1/113]). Mean adjusted HIVTSQs scores improved significantly (p< 0.001) from BL to M12 for LA (+3.40) vs. BIC/FTC/TAF (–1.07) participants.

**Figure:**
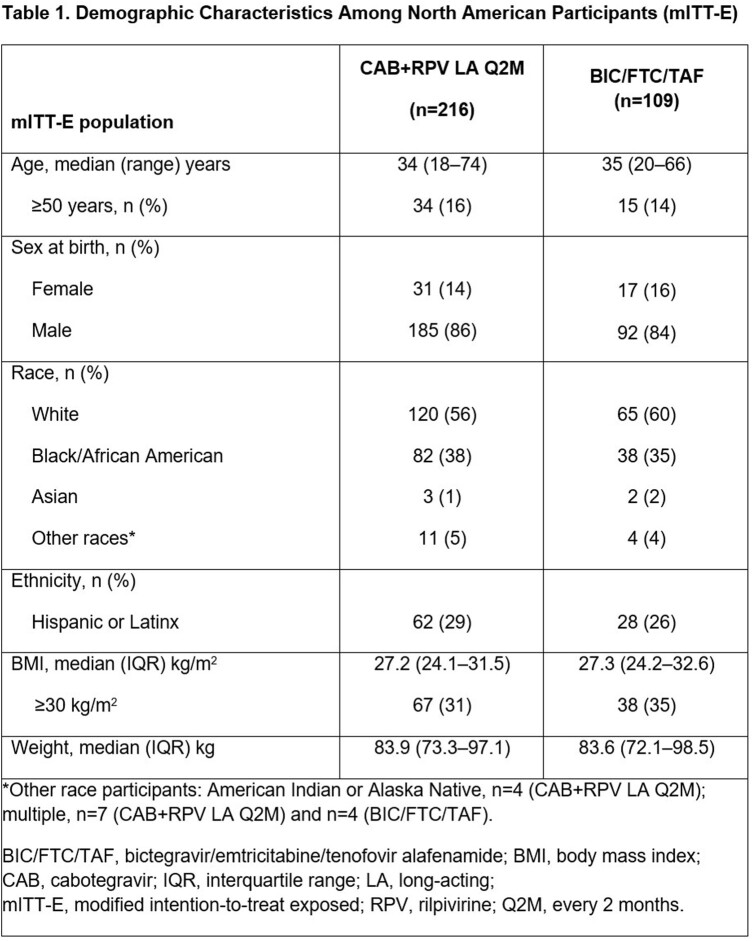

**Figure:**
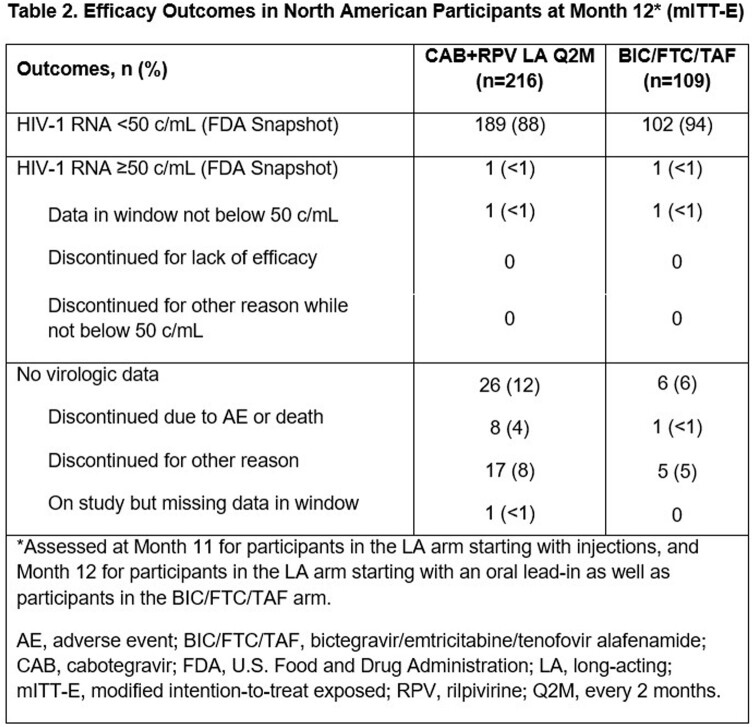

**Conclusion:**

Consistent with the overall SOLAR study population, switching to CAB+RPV LA from BIC/FTC/TAF was efficacious and well tolerated, with improved treatment satisfaction, in NA participants.

**Disclosures:**

**Mehri McKellar, MD**, Gilead Sciences, Inc: Grant/Research Support **Paula Teichner, PharmD**, GlaxoSmithKline: Stocks/Bonds|ViiV Healthcare: Employment **Jonathan Angel, MD**, Gilead: Advisor/Consultant|Gilead: Grant/Research Support|Gilead: Honoraria|Merck: Grant/Research Support|ViiV Canada: Advisor/Consultant|ViiV Canada: Grant/Research Support|ViiV Canada: Honoraria **Lori A. Gordon, PharmD**, ViiV Healthcare: Stocks/Bonds **Kenneth Sutton, MA**, ViiV Healthcare: Employment|ViiV Healthcare: Stocks/Bonds **Denise Sutherland-Phillips, MD**, ViiV Healthcare: Employment|ViiV Healthcare: Stocks/Bonds **Christine L. Talarico, M.S.**, ViiV Healthcare: Stocks/Bonds **Rimgaile Urbaityte, MSc**, GSK: Employment|GSK: Stocks/Bonds **Rodica Van Solingen-Ristea, MD**, Janssen R&D: Employee **Bryan Baugh, MD**, Johnson & Johnson: Stocks/Bonds **Ronald D'Amico, DO, MSc**, ViiV Healthcare Ltd: Stocks/Bonds **Jean A. van Wyk, MBChB, MFPM**, ViiV Healthcare Ltd: Stocks/Bonds

